# Towards an Ecological Understanding of Dinoflagellate Cyst Functions

**DOI:** 10.3390/microorganisms2010011

**Published:** 2014-01-03

**Authors:** Isabel Bravo, Rosa Isabel Figueroa

**Affiliations:** 1 Spanish Oceanographic Institute, Subida a Radio Faro 50, Vigo 36390, Spain; E-Mail: rosa.figueroa@biol.lu.se; 2 Aquatic Ecology, Department of Biology, Lund University, Lund 22362, Sweden

**Keywords:** cysts, dinoflagellate life cycle strategy, dinoflagellate reproduction, resting cysts, pellicle cysts

## Abstract

The life cycle of many dinoflagellates includes at least one nonflagellated benthic stage (cyst). In the literature, the different types of dinoflagellate cysts are mainly defined based on morphological (number and type of layers in the cell wall) and functional (long- or short-term endurance) differences. These characteristics were initially thought to clearly distinguish pellicle (thin-walled) cysts from resting (double-walled) dinoflagellate cysts. The former were considered short-term (temporal) and the latter long-term (resting) cysts. However, during the last two decades further knowledge has highlighted the great intricacy of dinoflagellate life histories, the ecological significance of cyst stages, and the need to clarify the functional and morphological complexities of the different cyst types. Here we review and, when necessary, redefine the concepts of resting and pellicle cysts, examining both their structural and their functional characteristics in the context of the life cycle strategies of several dinoflagellate species.

## 1. Introduction

**Figure 1:**
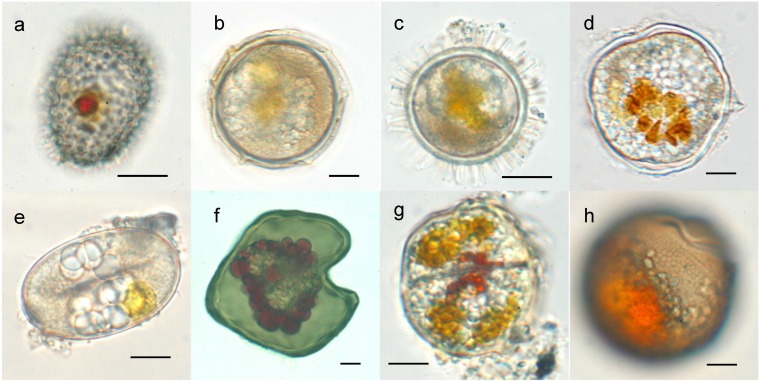
Resting cysts of *Scripsiella* sp. (**a**), *Alexandrium pseudogoniaulax* (**b**), *Protoceratium reticulatum* (**c**), *A. taylori* (**d**), *A. tamarense* (**e**), *Protoperidinium oblongum* (**f**), *Kryptoperidinium foliaceum* (**g**), and *Gymnodinium catenatum* (**h**). Scale bar: 10 µm.

Dinoflagellates are aquatic, highly ecologically diverse, eukaryotic organisms. As one of the major groups of phytoplankton, they are important contributors to aquatic primary production. Because many dinoflagellate species are associated with harmful algal blooms (HABs) they have been the focus of intense study, including of their complex life cycle. More than 10% of the approximately 2000 known marine dinoflagellate species produce cysts as part of their life cycle (Figure 1). These benthic phases play an important role in the ecology of the species, as part of a planktonic-benthic link in which the cysts remain in the sediment layer during conditions unfavorable for vegetative growth and, from there, reinoculate the water column when favorable conditions are restored. 

Indeed, during dinoflagellate evolution the need to adapt to fluctuating environments and/or to seasonality is thought to have driven the development of this life cycle stage. Most protists form dormant cysts in order to withstand starvation and UV damage [1]. However, there are enormous differences in the main phenotypic, physiological and resistance properties of each dinoflagellate species cysts. Unlike in higher plants most of this variability, for example in dormancy periods, has not been proven yet to be attributed to latitude adaptation or to depend on other life cycle traits [2,3]. Thus, despite recent advances in our understanding of the life histories of many dinoflagellate species, including the role of cyst stages, many gaps remain in our knowledge about their origin and functionality. 

Recognition of the capacity of dinoflagellates to encyst dates back to the early 20th century, in biostratigraphic studies of fossil dinoflagellate cysts. Reinsch [4] was the first to identify cysts as the fossilized remains of dinoflagellates. Unfortunately, his observations were overlooked and the first papers to gain notice were published in the 1940s and 1950s, when encystment by living dinoflagellates was described [5,6,7,8]. Later, cyst formation from gamete fusion was reported, which led those authors to conclude that encystment is associated with sexual reproduction [9,10,11,12]. These observations also gave credence to the idea that microalgal encystment is essentially a process whereby zygotes prepare themselves for a dormant period [13]. Because the resting cysts studied until that time came from sexual processes, dormancy was associated with sexuality, a presumption that was maintained for many years. This attribution was coincident with evolutionary theories about the origin of eukaryotic cell fusion and sexuality, which postulated advantages for species with diploid resting stages, in their ability to withstand nutrient stress and mutational UV radiation through recombinational repair, and for those with haploid vegetative stages, as asexual division doubles the number of cells [1]. Nonetheless, certain environmental conditions may limit the advantages of recombination and sexuality [14], such that in fungi, for example, complex combinations of haploid and diploid cycles have evolved that include asexual and sexual resting stages [15].

However, in the general life cycle of cyst-producing dinoflagellates as outlined in the 1960s and 1970s, resting cysts were assumed to be the fate of sexuality [12,16,17,18], which itself was regarded as a response to stress or unfavorable conditions. Sexuality involves the fusion of haploid gametes from motile planktonic vegetative stages to produce diploid planozygotes that eventually form cysts, or hypnozygotes, whose germination is subject to both endogenous and exogenous controls. Endogenously, a species-specific physiological maturation minimum period (dormancy) is mandatory before germination can occur. Thus, hypnozygotes were also referred to as “resting” or “resistant” cysts, in reference to this physiological trait and their capacity following dormancy to remain viable in the sediments for long periods of time. Exogenously, germination is only possible within a window of favorable environmental conditions. 

Yet, with the discovery that planozygotes were also able to divide it became apparent that the complexity of dinoflagellate life cycles was greater than originally thought [19,20]. Following corroboration of this behavior in several species, the capacity of dinoflagellate sexual phases to restore the vegetative phase, bypassing cyst formation, became well accepted [21,22,23,24]. Furthermore, Kremp and Parrow (2006) [25] showed that the dormant resting cysts of the Baltic cold water dinoflagellates *Scrippsiella hangoei* and *Gymnodinium* sp. were formed by the direct encystment of haploid vegetative cells, *i.e.*, asexually. In addition, for the zygotic cysts of *Pfiesteria piscicida* dormancy was not essential [26].

These studies opened up two lines of discussion: (1) the previously unrecognized importance of asexual cysts in dinoflagellate survival and (2) the difficulties in discriminating between sexual and asexual cyst morphologies. Both are closely related to another topic that has been discussed during the last 20 years: the nature and relevance of thin-walled non-dormant cysts. These cysts were referred to as thin-walled cysts by Fritsch in 1935 but have been also called “ecdysal”, “pellicle”, or “temporary” cysts because of their ecdysal origin, pellicle-layer wall, and absence of dormancy [18,27,28,29]. According to Dale (1983) [30], these synonymous or overlapping terms implied the asexual origin of these cysts and their short duration. Both Anderson and Wall (1978) [18] and Dale (1983) [30] viewed these thin-walled cysts as unlikely to play a significant role in initiating toxic blooms; on the contrary, they were thought to be a product of cell ecdysis in response to stress. Thus far, at least 48 species are known to form thin-walled cysts as part of their life cycle. Their formation has been detected under very different conditions, in both culture and in nature [31]. As the need for more appropriate terminology to describe thin-walled non-dormant cysts became apparent, the term “pellicle cyst” was adopted [31]. It is now well established that pellicle cysts are the product of asexual or sexual reproduction and that they indeed play a significant role in dinoflagellate bloom dynamics [12,26,32,33].

**Figure 2:**
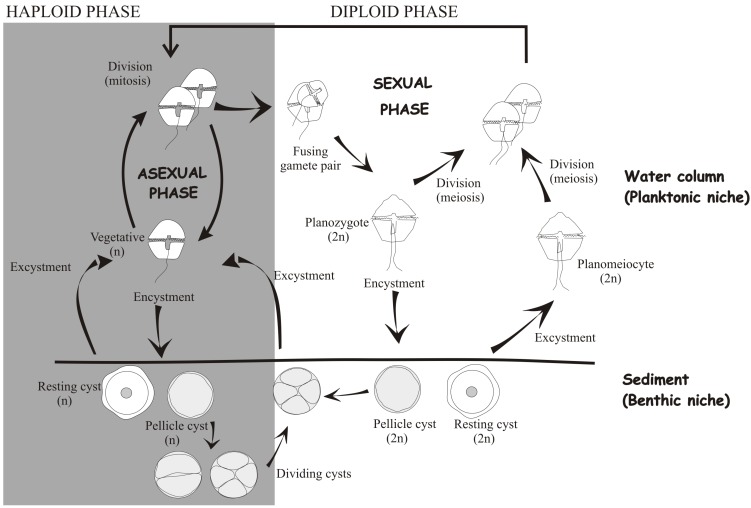
The life cycle of dinoflagellates, including all possible described transitions.

Another type of dinoflagellate cyst that deserves consideration comprises the dividing non-motile stages, or so-called division cysts, of a few dinoflagellate species, *i.e.*, *Woloszynskia apiculata* (transferred to the genus *Tovellia* by [34]), *Alexandrium taylori*, *Protoperidinium steidingerae*, *P. depressum*, *Kryptoperidinium foliaceum*, and *Pfisteria piscicida*. Based on their morphology, division cysts should be considered pellicle cysts [12,23,26,35,36,37], although in *Vulcanodinium rugosum* this assignment is not clear [38]. Cyst division seems to be relatively common among dinoflagellates. The flagellated cells shed their flagella and theca, round up, and sink, subsequently forming cysts that undergo division within minutes to hours after encystment. In these cysts, germination consists of division yielding two or more cells, depending on the species. The asexual and sexual origins of division cysts are depicted in Figure 2. Given the great difficulty of differentiating vegetative cells from planozygotes along with the fact that planozygote division remains poorly understood, it is quite likely that sexual cyst division is more frequent than previously acknowledged.

Recent, further recognition of the complexity of dinoflagellate life cycles has underlined the need to expand our knowledge of their many variations. Here we offer a review and discussion of the structural features and functions of resting cysts *versus* pellicle cysts. Because the main traits used in past and present cyst studies to classify cysts are related to characteristics of the cell wall, we use the term “pellicle cysts” to refer to thin-walled cysts, and “resting cysts” for cysts classified as thick-walled, aiming to be generic and thereby to facilitate literature comparisons. 

## 2. Structural Features of Dinoflagellate Cysts

The morphology of resting cysts was well defined in the 1970s and 1980s, and, as noted above, in earlier paleontological studies of fossil cysts. Resting stages differ greatly from motile stages with respect to morphology, such that a species description is completed when the characteristics of the two are compared. Generally, cysts are defined on the basis of their shape and color, the structure and surface ornamentation of the wall, and the features of the archeopyle, paratabulation, and cell contents. These characteristics are for the most part distinctive and constant within a species [30] and are well defined in both the paleontological [39,40,41,42,43] and planktonological [30,44,45] literature. However, for the cysts of some species, morphological differences related to differences in environmental conditions have been observed, as discussed below for *Lingulodinium polyedrum* [46,47].

### 2.1. Cyst Wall Structure

The structure and composition of the cyst wall are among the most distinctive traits of dinoflagellate cysts. The absence or presence of a thick wall differentiates pellicle cysts from resting cysts. Whereas the wall of resting cysts is formed by one, two or three layers and is thus moderately thick to very thick, the thin wall of pellicle cysts derives from the pellicle layer of the motile stage [30]. However, neither the detailed structure of the cyst wall nor the process involved in its formation is well understood. 

Paleontologists studying fossil dinoflagellates were the first to recognize the physical and chemical resistance of resting cyst walls. The multilayer wall was initially thought to be composed of cellulose or sporopollenin, a still poorly defined, highly resistant, organic polymer found in spores and pollen grains [48,49]. However, chemical studies questioned the presence of sporopollenin as a major component of dinoflagellate resting cyst walls [50,51]. Its definitive chemical structure remains to be resolved since it was partly defined from fossilized spores and pollen walls whereas it is now clear that the structural changes in those preserved materials during burial and diagenesis have been underestimated [52]. Recent studies reported that the walls of extant dinoflagellate resting cysts are different from those of other algae as the former contain dinosporin, which is resistant to chemical degradation and undergoes scant biodegradion, as evidenced by the rich fossil record of dinoflagellate cysts. Yet, the structure of dinosporin remains a matter of discussion [50,53]. Its complex macromolecule structure was initially thought to consist of aliphatic and aromatic moieties [50], with the latter acting as a protective agent. However, recent analysis of *L. polyedrum* suggests that dinosporin is not aromatic and is most likely carbohydrate-based. As such, it is much more closely related to cellulose than to sporollenin or algaenan, the resistant biopolymer widespread in green algae [53]. Yet, it is unclear whether the variability in dinoflagellate life cycles is reflected in differences in the chemical composition of the cyst wall. 

To clarify the differences between the walls of pellicle cysts and resting cysts requires detailed insights into the processes involved in their formation. Pellicle cysts are non-motile cells surrounded by a membrane and a thin continuous wall, both of which are formed by motile cells that shed their theca and outer amphiesma [54]. However, whether the pellicular layer of the resulting cyst is already present in the motile cell beneath the theca is unclear. Loeblich [55] recognized the pellicle as a continuous fibrous layer lying internal to the vesicular layer of the amphiesma. The pellicular layer was shown to vary in thickness and composition among several dinoflagellates, since only half the pellicles tested were resistant to acetolysis [56]. Taylor [54] also reported a pellicular layer in the motile phase of the genus *Achradina*. In these and other taxa, the pellicular layer was the principal strengthening element of the periphery of the motile stage cell. By contrast, Höhfeld and Melkonian [57] restricted the use of the term “pellicle” to the covering of ecdysed cells and noted alterations in the pellicular layer of the vegetative cells of some dinoflagellate species during ecdysis. Those authors offered a revised model of pellicle formation during ecdysis in which several amphiesmal components are shed: the plasma membrane, the outer amphiesmal vesicle membrane and, in thecate dinoflagellates, the thecal plates. The inner membranes of the amphiesmal vesicles then fuse to form a continuous membrane (the pellicle membrane) that remains tightly appressed to an underlying amorphous layer (the pellicular layer). That study also described mature pellicle cysts with a significant thick pellicular layer that becomes the most prominent portion of the pellicle. However, it was claimed that the pellicular layer does not increase in thickness upon ecdysis in all dinoflagellates and that in the vegetative cells of some species it may even be already present rather than induced by ecdysis. In summary, the observations of Morrill and Loeblich [56], in their histochemical study of pellicles in 45 dinoflagellate taxa, and the differences in the structures of pellicle cysts described by Höhfeld and Melkonian [57] together suggest that in both motile cells and the cysts of the different taxa pellicle resistance is highly variable and depends on cyst wall structure and composition. 

Unlike encystment, which has been examined in many dinoflagellates, the formation of the resting cyst wall has been scarcely investigated. Kokinos and Anderson (1995) [47] provided a detailed optical microscopy description of the encystment process of *L. polyedrum*, in which three important features were recognized: (1) expansion of the outer membrane, which along with the dissociating theca pulls away from the cell body, encircles it and gives the encysting cell the appearance of an inflating balloon; (2) cytoplasmic surface globules that radially extend outwards beneath the dissociating theca to form the processes of the resting cysts; and (3) rupture of the expanding outer membrane before resting cyst development is completed, which accounts for the size and morphological variations in the cyst processes. Together, they provide evidence that key events in cell wall development in the resting cyst take place within the expanding interstice between the outer membrane and the cytoplasm. In addition, morphological variations in the cysts depend on the extent of developmental progression before the expanding outer membrane ruptures. Nonetheless, whether this pattern of cyst wall development is common in dinoflagellates has yet to be confirmed because of the very limited number of descriptions in the literature. Swelling of the outer membrane and the formation of an interstice around the cytoplasm where the new wall is formed were also described for *Gymnodinium pseudopalustre* [12]. This peripheral interstice may indicate the consolidation and/or modification of cytoplasmic components in preparation for cyst formation. While it may not be unique to planozygotes, its presence around the cytoplasm of vegetative cells preparing for ecdysis has been suggested [47]. 

In the literature on resting cyst formation, the most commonly reported characteristic is the deposition of one or more new wall layers surrounding the cytoplasm. In armored species, these layers typically form beneath the theca. However the dissociation of the theca surrounding the cell during cyst wall formation seen in *L. polyedrum* [47] does not occur in all dinoflagellates. For example, in the planozygotes of *Scripsiella* sp. the theca is shed at a very early stage of encystment, before wall formation [58] although during this stage there are already three membranes, suggesting that they formed concomitantly with the cellular preparations for ecdysis. This indicates that the preliminary membranes of the cysts form when the cells are preparing for ecdysis, which coincides with the above-mentioned pellicle cysts of Höhfeld and Melkonian [57]. Although the process varies between species, at least in some dinoflagellates ecdysis seems to be a key process in the development of the walls of both pellicle and resting cysts. 

Ultrastructural studies of the encystment of a large number of species have revealed differences in the wall structure of the different types of cysts and between newly formed and mature hypnozygotes [48,59,60]. A three-layered wall seems to be common to many cyst types, although the structure and thickness of the cyst wall seem to vary and may be a function of maturation [61]. A few studies reported that in early cyst stages a less dense material initially underlies the pellicular electrodense layer and becomes thicker during maturation [59,60]. Other studies reported the deposition during encystment of mucofibrillar or granular material in some of the layers of the triple membrane of *Scripsiella* sp. and *Woloszynskia tylota* (transferred to the genus *Tovellia* by [34]) [48,58]. Despite these structural differences, encystment seems to include a gradual process of cyst wall formation in which the cells (planozygotes in the case of sexual cells) ecdyse from the theca, with the pellicular layer forming the outer layer and the other layers then forming below it. This process shares several properties with pellicle formation in pellicle cysts (see references above) and suggests that differences in resistance can be ascribed to the thickness and exact composition of the layers of the cyst wall. 

### 2.2. Cytoplasmic Features

**Figure 3:**
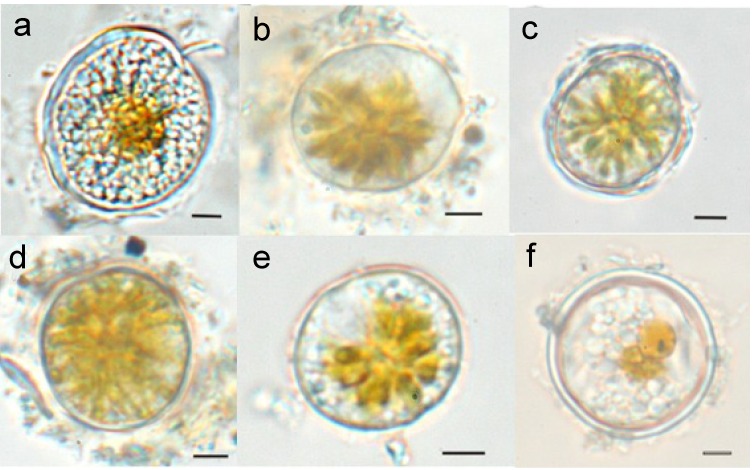
Resting cysts and pellicle cysts of *Alexandrium minutum*. Resting cyst from a sediment trap (**a**), pellicle cyst with only a thin pellicle layer (**b**), pellicle cyst with the theca of the vegetative cell remaining (**c**), pellicle cyst with uncondensed cytoplasm (**d**), pellicle cyst with condensed cytoplasm (**e**), and double-walled resting cyst from the sediment (**f**). Scale bar: 10 µm. Adapted from [31].

In addition to the changes in the cyst wall, metabolic changes during encystment have been described. These occur in association with the breakdown of the cell’s metabolic machinery; particularly that involved in photosynthesis. The most obvious transformations are an increase in the degree of thylakoid stacking, the accumulation of lipids in the plastid, the formation of a large, orange-brown accumulation body, the disappearance of Golgi bodies, and an increase in the number of vacuoles together with an internal accumulation of polyhedral bodies [48,59]. These changes are responsible for the distinctive cytoplasmic features of resting cysts, which are mostly lighter in color and more granular in content than mobile planozygotes or pellicle cysts. However, whether these changes are a general trait of dinoflagellates is not clear, given the scant literature on this topic. The accumulation body, formed by the concentration of carotenoid pigments [13], and its coloration have also been described as distinctive features of the resting cysts of some species, *i.e.*, *Alexandrium* spp. [62]. Bravo *et al*. (2010) [31] noted differences in the wall thickness, cytoplasmic content, and dormancy period of the resting, pellicle and thecate cysts of *Alexandrium minutum* (Figure 3). The accumulation body was condensed only in dormant resting cysts and the cytoplasmic appearance of non-dormant pellicle and thecate cysts varied, ranging from being similar to vegetative cells to a more compressed star-shaped arrangement of the chloroplasts in the cell center. These observations suggest that the transformations of plastid structures are greater for resting cysts than for pellicle cysts, as is also the case for the structure of the cyst wall. 

### 2.3. Environmental Influences

Although much remains to be learned about the process of encystment, as far as it is now known dinoflagellates resting cysts have a more resistant wall than pellicle cysts. But what factors contribute to this resistance? Variations in salinity and a combination of salinity and temperature are known to influence cyst morphology in some dinoflagellate species, mainly by affecting the length of the cyst processes. For the resting cysts of *L. polyedrum* and *Protoceratium reticulatum*, this relationship between environmental conditions and morphology was suggested as a very useful proxy for salinity in paleoclimate studies [46,63]. A strong correlation between cyst-process length and salinity has also been described for the cysts of *Gonyaulax baltica* [64], but not for those of *Pyrophacus steinii* [65]. However, cysts of both these species as well as those of *P. steinii* were shown to undergo morphological variations in response to changing temperatures [64,65,66].

**Figure 4:**
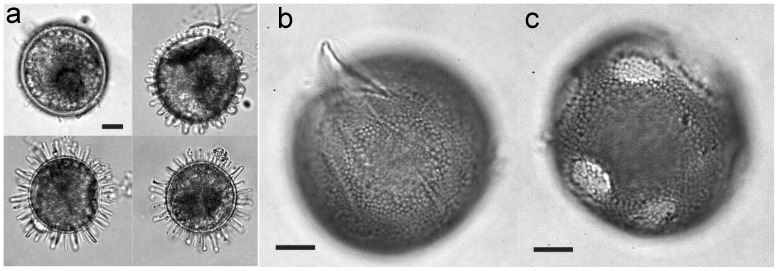
Differences in cyst wall development during the encystment of *Lingulodinium polyedrum* (**a**) and *Gymnodinium catenatum* (**b**,**c**). Scale bar: 10 µm. Adapted from [22] and [24].

Differences in cyst spine sizes (Figure 4a), associated with different encystment times and therefore probably with differences in nutrient status, were observed in cultures of *L. polyedrum* [22]. In that species and in *Alexandrium taylori*, the formation of sexual pellicle cysts and resting cysts was shown to be dependent on the nutritional content of the external medium [22,23]. The data from those studies suggest that P and N levels determine the type of cyst produced during reproduction: pellicle cysts under phosphate-limited conditions and resting cysts during nitrate limitation. In cultures of *Gymnodinium catenatum*, N limitation alters formation of the cyst shell, as holes were seen in the external wall and in some cysts the external layer was completely lacking (Figure 4b,c) [24]. Interestingly, germination and viability were the same in these cysts as in normal-walled cysts, although the long-term viability of the defective cysts was not examined. 

## 3. Functions of Dinoflagellate Cysts

Among the main functions of dinoflagellate cysts are nuclear replenishment and recombination through meiosis (in the case of sexual cysts), protection, propagation, and dispersion. These functions are important in bloom dynamics, initiation, and termination and in allowing cellular adaptation and survival during adverse conditions [67,68]. 

Although meiosis was first ascribed to resting cysts, it has since been recognized in planozygotes and sexual pellicle cysts. Furthermore, the observation in two dinoflagellate species of asexual resting cysts [25] suggests that resting cysts are not the only fate of sexuality and that it cannot be unequivocally associated with resistance to unfavorable conditions. Thus, the sexual or asexual origin of each cyst type should be carefully studied and hypnozygotes and resting cysts should no longer be considered equivalent. 

In fact, reports over the last 10 years suggest that, in general, there is no landmark of cyst sexuality, neither morphological nor life-strategy-related; instead, each species seems to follow its own defined pattern. What is clear is that sexual cyst formation accounts for just a fraction of the total sexual reproduction. In cultures, environmental conditions regulate life cycle transitions and commit planozygotes to either divide or become cysts. Microscopy observations have demonstrated nuclear cyclosis, *i.e.*, the rapid rotation of the nucleus within the cell [69,70], considered indicative of meiosis [10,12]. Nuclear cyclosis has been observed in mobile planozygotes, resting cysts, and after cyst germination [10,12,26,35,71]. Inheritance studies support the notion that genetic recombination occurs during planozygote [72] or planomeiocyte (diploid germling) division [73], the exception being a report of cyclosis in vegetative stages of *Paulsenella* [74]. Accordingly, the results of genetic studies are consistent with the conclusion that resting sexual cysts constitute a species reservoir of genetic diversity (e.g., [75]), although the relative contribution of each meiotic route to the genetic structure of the population remains to be determined.

### 3.1. Survival during Unfavorable Conditions

Protection, propagation, and dispersion are profoundly related to the survival time of cysts exposed to unfavorable environmental conditions. Protective attributes favor longer survival and therefore increase the likelihood of propagation and dispersion. Because of their strong, thick wall and the above-mentioned metabolic changes, resting cysts have been traditionally viewed as a life cycle stage that allows the dinoflagellate to withstand unfavorable conditions and is thus related to long-term survival. Indeed, the longest-surviving dinoflagellate cysts were in the form of resting cysts, with the survival periods of various marine dinoflagellate resting stages ranging from several months to 100 years [76]. In the following, we summarize the evidence in the literature supporting encystment as an advantageous adaptation to unfavorable conditions and as a survival strategy for cyst-producing dinoflagellate species. When possible, the differences between the different types of cysts are highlighted. 

#### 3.1.1. Resting Cyst Formation as a Strategy for Withstanding Unfavorable Temperature and Nutrient Conditions

Dinoflagellate encystment is generally considered a response to the stress imposed by suboptimal growth conditions [77]. Among all environmental factors, nutrient limitation and changes in temperature are the most common triggers for resting cyst formation [12,24,78,79,80]. By contrast, fewer studies have related temperature and nutrient limitation to pellicle cyst formation. In their study of *Prorocentrum minimum*, Grzebyk *et al*. (1996) [81] reported high percentages of pellicle cysts in response to temperature stress. Similarly, pellicle cyst formation by the *Alexandrium catenella* in response to phosphorous limitation was reported [82]. 

#### 3.1.2. Resting Cyst Formation as a Protective Strategy during Anoxia and Darkness

Resting cysts are frequently buried below the sediment surface, and thus subjected to both darkness and low oxygen conditions. This observation has motivated studies of the effects of anoxia and light conditions on cyst survival. Anderson *et al*. (1987) [83] demonstrated that resting cyst germination was equally inhibited by anoxia in six dinoflagellate species, albeit with clear differences in their responses to darkness or to different light levels. In general, cyst germination in response to darkness seems to vary depending on the species [83,84,85] whereas anoxia may be the most important factor preventing germination since it results in complete inhibition rather than merely a delay, as occurs with light deprivation [83]. These results would explain the accumulation of large numbers of living cysts below the surface of marine sediments and implies that encystment is a protective mechanism that facilitates the eventual reinoculation of the respective species into the plankton when resuspension events return buried cysts to the surface. However, in the absence of these events or of other advective processes, relatively few cysts are able to germinate. The importance of anoxia in sustaining the resting cyst stage is supported by dormancy studies, in which anoxic conditions were used to maintain long-term cyst viability [72,80]. For pellicle cysts, similar investigations into the effect of anoxia are for the most part lacking. The formation of pellicle cysts as a strategy to cope with darkness has been reported for species of the genus *Scrippsiella* [86], in which respiratory rates in the dark were reduced to very low levels [86,87].

#### 3.1.3. Resting Cyst Formation as a Strategy to Avoid Grazing

Dinoflagellate encystment may also be a predator-avoidance adaptation induced by chemical cues that signal the presence of zooplankton. For *Peridinium aciculiferum*, it would explain the lower rates of resting cyst germination in the presence of potential grazers [80]. The same study also suggested that dinoflagellate cysts are able to detect chemical signals emitted by predators. However, in another report, there was no apparent influence of the copepod *Acartia tonsa* on the formation of pellicle cysts by *Scrippsiella trochoidea* [86]. In this dinoflagellate species, allelopathic effects on pellicle cyst formation may provide a defensive response to deleterious chemical cues [88]. Pellicle cysts were observed to readily revert to a motile stage after a time-dependent degradation or inactivation of the allelochemicals produced by *Alexandrium ostenfeldii* [89].

Safe passage through the digestive apparatus of a predator also ensures survival. However, the fate of cysts ingested by planktonic and benthic grazers is unknown. Resting cysts of *S. trochoidea* survive after ingestion by copepods, although subsequent germination success depends on the grazer species [90]. The survival of pellicle cysts after passage through the digestive tract of *Crassostrea gigas* has been reported [91].

#### 3.1.4. Resting Cyst Formation as a Defense against Parasitic Attack

The formation of both resting and pellicle cysts confers protection against parasitic attack. In cultures of *A. ostenfeldii*, pellicle cyst formation increased in the presence of the parasite *Parvilucifera* [92], although the pellicle cysts of *A. minutum* eventually became infected and only resting cysts were finally resistant to this parasite [93]. Thus, pellicle cyst formation may be an alternative, short-term solution if sexuality is not possible, either because of the absence of a partner or for cells unable to mate. Resting cysts could, however, carry latent parasite stages that become active after germination. For example, the parasite *Amoebophrya* sp. caused the accelerated production of *S. trochoidea* resting cysts, as the only stage resistant to infection. However, infective cycles in the absence of the parasite were observed after resting cyst germination, consistent with the simultaneous dormancy of the parasite and its dinoflagellate host [94].

### 3.2. Long-Term *versus* Short-Term Survival

A long-term survival function was proposed for the resting cysts of *Gonyaulax tamarensis* (now *A. tamarense*) as part of the “cyst hypothesis”, according to which resting cysts were considered to enable overwintering of the population in sediments. The protection conferred by resting cysts promotes the annual recurrence, propagation, and dispersion of blooms [18,95]. The dormancy requirement, attributed exclusively to resting cysts in contrast to non-dormant, fast-germinating pellicle cysts (therefore copiously referred to in the literature as “temporary cysts”), supports this stage as crucial to the long-term survival of the species. 

Dormancy is defined as the period in an organism’s life cycle when growth, development, and physical activity are temporarily suspended by active endogenous inhibition. Thus, even under favorable conditions, dormant cysts are not able to germinate until the dormancy period is completed [96]. Accordingly, dormancy is an important stage in the life history of a species, one that strongly determines bloom seasonality [80,97,98]. However, the relationship between this endogenous clock and seasonality is not well understood and there are very few cases in which it has been confirmed. The dormancy features of dinoflagellate cysts vary according to environmental conditions, such as temperature and nutrient levels [82,99], and the amounts of intracellular storage products may influence the duration of this mandatory resting stage [100]. 

The massive cyst formation that follows the spring dinoflagellate bloom in the Baltic Sea is a good example of how resting cysts provide a sedimentary “seed” bank from which the plankton is repopulated in subsequent seasons. In the Baltic Sea, the very large and widely distributed populations of benthic resting cysts that overwinter in its sediments guarantee the survival of vernal-bloom-forming dinoflagellates, including *Peridiniella catenata*, *Bicheleria baltica*, *Gymnodinium corollarium*, and *Woloszynskia* spp. [101,102,103]. Since the early 1980s, spring blooms of these cold-water dinoflagellates have become a recurrent phenomenon, in which resting cyst formation is an important contributing factor. 

The high survival and growth rates of the dinoflagellate *Pentapharsodinium dalei* from resting cysts germinated from a century-old sediment further support the notion of seed banks as a source of phytoplankton reserves [104,105]. The viability of resting cysts for over 40 years was similarly demonstrated for other species (*L. polyedrum*, *Scrippsiella* spp., and *Prorocentrum reticulatum*) and is considered likely for more than five other species whose cysts were found in ~90-year-old sediments [104]. Thus, the resting stages of dinoflagellates share features with other primary producers, *i.e.*, diatom resting stages, capable of long-term survival and regarded as important contributors to the resilience and diversification of marine life throughout the history of the Earth [105]. 

The role of dinoflagellate resting cysts as an adaptation allowing long-term survival was recently reviewed by Smayda and Trainer (2010) for upwelling systems [106]. The authors cited the difficulty of proving a survival-seeding strategy based on resting cysts because of the abundant contradictions between resting-cyst and vegetative-cell dynamics not only in upwelling systems but also in other environments. These discrepancies suggest that planktonic *versus* benthic dinoflagellate stages reflect the interplay of intricate biotic and abiotic factors, many of which are as yet unknown. Yet the authors acknowledged the long-term survival function of resting cyst in species with a high encystment:excystment ratio, such as occur in sediments with an intense accumulation of resting cyst banks. Where seed banks persist in the sediment for many years, resting cyst germination might explain the sudden re-appearance of a bloom after decades in which none occurred. 

Despite the short-term survival of pellicle cysts, their contribution to dinoflagellate species propagation is likely to be an important one, as demonstrated for the bloom dynamics of *Alexandrium taylori*. The benthic and planktonic phases of this dinoflagellate are tightly linked, with their exchange taking place daily. In this life strategy, immobile cells develop that spend the night deposited on the seabed and are able to return to the planktonic phase a few hours later [33,107]. These pellicle cysts have been considered both as temporary cysts, because of their relatively brief duration, and as ecdysal pellicle cysts, because the non-motile cells are formed by ecdysis [23]. They are also recognized as dividing cysts that undergo division in the benthic stage (Figure 2) to yield two, four, or six mobile cells [23]. Encystment in *A. taylori* seems to be aimed at minimizing advective losses due to breeze-forced coastal flows [108]. Thus, the propagation of this species is guaranteed by its ability to form non-dormant cysts that ensure the persistence of a motile stage in a fluctuating environment. 

Nonetheless, there are also examples of thick-walled cysts associated with functions other than long-term survival. The complex sexual life cycle of *Gymnodinium catenatum* includes sexual stages and the avoidance or shortening of a thick-walled cyst stage depending on external conditions [24]. Blooms of this species in the NW Iberian Peninsula are not directly caused by resting cyst germination because they are not linked to cyst seed beds but rely on advected vegetative populations to the coasts during the relaxation of coastal upwelling [109]. This bloom mode corresponds well with the life cycle characteristics of *G. catenatum* because the dormancy period of its thick-walled cyst may be as short as 6 days [110]. Because of their short dormancy period, and the ease with which excystment occurs, the majority of *G. catenatum* cysts from the sediment germinate before reaching bloom-promoting conditions [109]. Although it cannot be ruled out that resting cysts of this species periodically inoculate the water column to a limited extent, its bloom strategy in NW Iberian Peninsula waters is not the same as proposed by Anderson and Wall (“the cyst hypothesis”) for *G. tamarensis* (now *A. tamarense*) [18].

Another interesting question is whether pellicle cysts play a role in long-term survival. The pellicle cysts of some species (e.g., *Cochlodinium polykrikoides*, *Scripsiella hangoei*, and *Ostreopsis* cf. *ovata*) have been definitively shown to remain viable for months under unfavorable conditions and are good examples of the dual functionality of cysts: that is, their ability to quickly assume a motile stage but also to survive for prolonged periods in the dark as non-motile stages [87,111,112]. This suggests the ability of these cysts to overcome adverse conditions in nature, giving rise to a bloom when conditions are again favorable. Further studies of pellicle cysts are needed before their survival traits and their role in species dynamics are completely understood. 

Also of interest are the non-flagellated vegetative-like cells identified in cultures and in field studies. These so-called thecate cysts survive in this form for a variable period of time, ranging from days, in the case of *A. minutum*, to months in the case of *O.* cf. *ovata* and *Prorocentrum lima*, and then germinate [31,112,113].

### 3.3. Life-Cycle Strategy and Habitat

Like other traits in nature, a life cycle strategy reflects the respective organism’s genotype and its tactical response to the environment [114]. Thus, the existence of one or another type of cyst and even the lack of cyst formation are key indicators of the ecological strategy of species. Accordingly, insights into the functions of the cysts can be acquired by relating them to the habitat in which they develop. Much more information is needed before the particular life history strategies of dinoflagellate species can be related to habitat characteristics, although some examples have already been mentioned in our discussion thus far.

The short-term pellicle cysts of *A. taylori* and *A. minutum*, two species that form blooms in nearshore coastal environments [108,109], have been associated with the avoidance of wind dispersion. The rapid interchange between planktonic and benthic forms minimizes advective losses of cells, facilitating bloom development. In addition, the larger the bloom, the larger the cyst bank on the seafloor, which guarantees the survival of the species. This strategy is clearly an adaptation to coastal habitats, such as those of the two aforementioned species, where the shallow waters allow the accumulation of cysts on the sea bottom. By contrast, in offshore environments, dormant resting cysts sink to the deep sea bottom, which probably rules out a survival strategy based on competitive seeding, since the cysts of these species would require frequent and strong resuspension events. In fact, some neritic species, such as upwelling dinoflagellates belonging to the genera *Ceratium*, *Dinophysis*, *Karenia*, and *Prorocentrum*, are holoplanktonic; that is, they do not form resting cysts [106]. 

There is very little information about the functions of division cysts, such as the pellicle cysts of *A. taylori*. The possibility of division, even by cells that have sunk and then settled in the sediments, “waiting” for the return of favorable conditions, is a clear adaptive advantage for dinoflagellate growth and constitutes an important advantage in the bloom dynamics of such species [33]. The blooms formed by *Alexandrium hiranoi* (cited as *Goniodoma pseudogoniaulax* by [32,115]) during high tides in rock pools along the Pacific coast of Japan are also thought to rely on the division of pellicle cysts, which ensure short-term survival during temporarily adverse conditions (dry at low tides). Furthermore, the long-term survival of that species is ensured by its ability to form double-walled resting cysts, which are dormant during the non-bloom season [32].

### 3.4. Dispersion

Dinoflagellate cysts promote species dispersion because they are transported by seeding within the sediment layer. Transportability in turn depends on the size, density, and settling velocity of the cyst [116], which implies differences in the dispersion properties of pellicle and resting cysts. While there is no information on pellicle cyst density, for the resting cysts of the species examined in one study differences of up to 0.01 g cm^−3^ were reported [68], with the lowest density (1.14 g cm^−3^) being that of the resting cysts of *Gymnodinium uncatenum*. However, whether the sinking rates, wall structure, and composition of the different cyst types are related to the ecological traits of the respective cyst-producing dinoflagellates has yet to be determined. 

Cyst ornamentation, in the form of spines and protuberances, is an important aspect of dinoflagellate dispersion even though its function is still under discussion. Presumably, these structures decrease the sinking rates because of the increase in drag, as described for planktonic organisms. However, spines can also promote sinking. For example the dense protruding short spines of *S. trochoidea* and other species do not alter cell resistance [117] but do increase cell density [116]. Furthermore, spines and protuberances can act to promote aggregation, which enhances the sinking rate [118]. Thus, in high-water-density environments, *L. polyedrum* processes are thought to increase the sinking rate of its cysts by favoring cluster formation [46]. In coastal semiconfined areas, cyst aggregation, such as observed in *A. catenella* or *A. minutum* but never in *G. catenatum*, may be an adaptation to promote cyst accumulation, which in turn promotes bloom recurrence. In the case of cysts that lack spines, aggregation is likely to be achieved by other methods, such as a mucilaginous coating. Furthermore, adherence to floating objects, whether by spines or a sticky surface, allows the crossing of eco-geographical barriers, thus supporting a genetic flux among apparently isolated populations [118]. For neritic species with a short dormancy period, such as *G. catenatum* in which the dormant stage is only 6 days, enhanced flotation increases the probability that the cyst will germinate before it reaches the ocean bottom [109]. The many cyst dispersion mechanisms highlight the need for further investigation before their influence on the survival of each species can be fully understood.

## 4. Future Perspectives

Much remains to be learned about the formation of pellicle and resting cysts, as even the differences in cyst structure and function in different species are not well defined. In fact, in some species it is difficult to determine whether they form resting or pellicle cysts because the structural characteristics of the cyst wall have yet to be unequivocally established, hindered by detections of intermediate forms [31]. The mechanisms regulating encystment and the extent to which pellicle and resting cyst formation represents different processes *vs*. the extremes of a developmental gradient controlled by as yet unidentified factors, are topics of great interest that merit further research. Furthermore, the life cycles of most dinoflagellate species are still poorly understood, with many gaps in our knowledge of those that have been described. It is therefore quite likely that, as these gaps are filled, an increasingly complicated range of encystment-related features will emerge. 

Following the earliest cyst studies, research efforts have largely focused on the morphological and structural characterization of cysts and of other stages involved in encystment and excystment, as well as on the nature of dormancy and the factors that regulate all of these processes. Full comprehension of the functions of cysts within the life cycle of the many dinoflagellate species requires studies not only on the role of benthic stages as a life-cycle strategy but also on the role of cysts within the cell cycle. Indeed, the cell-cycle-based control of encystment by *Alexandrium taylori* was suggested [23] to explain the different stages subsequent to planozygotes. Nutrient depletion was shown to affect cells in all phases of the division cycle (G1, S, or G2/M), specifically resulting in non-flagellated cells or pellicle cysts (considered representative of the G0 phase). The fate of the latter is either to persist as such, enter a new division cycle, or form long-term resting stages. That same study suggested that the formation of pellicle cysts or resting cysts is determined by a combination of internal and external factors that converge at a particular moment in the cell cycle and that the process is potentially reversible. Moreover, the relative quantity of some particular factor (e.g., nutrients) might tip the balance in favor of a particular type of encystment. 

Clearly, the differences underlying dinoflagellate species and the dynamics of their blooms will only be understood by identifying the determinants of encystment and how they interact to influence the cell cycle, both in sexual and asexual cells. The many open questions posed in this review will only be answered by continued research.
